# Development and external validation of prognostic scoring models for portal vein thrombosis: a multicenter retrospective study

**DOI:** 10.1186/s12959-023-00455-w

**Published:** 2023-01-23

**Authors:** Xuan Zhong, Shan Li, Jiali Hu, Jinlai Lu, Wei Wang, Miao Hu, Qinjuan Sun, Shuo Zhang, Xiaoqing Yang, Changqing Yang, Lan Zhong

**Affiliations:** 1 Present Address: Department of Gastroenterology, Shanghai East Hospital, Tongji University School of Medicine, 150, Jimo Road, Pudong New Area, Shanghai, 200120 China; 2 Present Address: Department of Gastroenterology, Shanghai Tongji Hospital, Tongji University School of Medicine, Shanghai, China

**Keywords:** Anticoagulation, Cox regression, Nomogram, Prediction model, Portal vein thrombosis

## Abstract

**Background:**

Portal vein thrombosis is a common complication of liver cirrhosis and hepatocellular carcinoma; however, few studies have reported its long-term clinical prognosis. This study aimed to establish and validate easy-to-use nomograms for predicting gastrointestinal bleeding, portal vein thrombosis resolution, and mortality of patients with portal vein thrombosis.

**Methods:**

This multicenter retrospective cohort study included 425 patients with portal vein thrombosis who were divided into training (*n* = 334) and validation (*n* = 91) sets. Prediction models were developed using multivariate Cox regression analysis and evaluated using the consistency index and calibration plots.

**Results:**

Predictors of gastrointestinal bleeding included a history of gastrointestinal bleeding, superior mesenteric vein thrombosis, red color sign observed during endoscopy, and hepatic encephalopathy. Meanwhile, predictors of resolution of portal vein thrombosis included a history of abdominal infection, C-reactive protein and hemoglobin levels, and intake of thrombolytics. Predictors of death included abdominal infection, abdominal surgery, aspartate aminotransferase level, hepatic encephalopathy, and ascites. All models had good discriminatory power and consistency. Anticoagulation therapy significantly increased the probability of thrombotic resolution without increasing the risk of bleeding or death.

**Conclusions:**

We successfully developed and validated three prediction models that can aid in the early evaluation and treatment of portal vein thrombosis.

**Supplementary Information:**

The online version contains supplementary material available at 10.1186/s12959-023-00455-w.

## Background

Portal vein thrombosis (PVT) is characterized by thrombosis in the portal vein or its branches, which can lead to portal hypertension and a series of pathophysiological changes [1–5]. Although the incidence of PVT in the general population is low (approximately 1%) [4, 6], the prevalence of PVT associated with liver cirrhosis ranges from 0.6 to 16% [3, 7]. Increased intrahepatic vascular resistance; reduced portal flow velocity; and methylenetetrahydrofolate reductase C677T, prothrombin G20210A, and factor V Leiden G1691A mutations are considered important risk factors for PVT in patients with cirrhotic livers [4]. Patients with non-cirrhotic livers also have risk factors for PVT, particularly those associated with a hypercoagulable state such as extrahepatic malignancies, myeloproliferative neoplasms, systemic lupus erythematosus, intra-abdominal infections, abdominal trauma, and intra-abdominal surgery (e.g., splenectomy) [6]. At present, the occurrence of PVT is unpredictable, and several aspects regarding its pathophysiology, prognosis, and treatment remain unknown.

Most patients with PVT do not show specific clinical manifestations and sometimes present with only mild abdominal pain, which is often ignored. In severe and persistent cases, PVT can cause liver injury, mesenteric vein embolism, intestinal perforation, gastrointestinal bleeding (GIB), recurrent thrombosis, and even death [8]. Therefore, early initiation of anticoagulation therapy is recommended for the treatment of PVT [1, 2]. However, recent studies have shown that spontaneous thrombotic recanalization can be achieved in 30–50% of PVT cases associated with cirrhosis and other etiologies [9]. Whether anticoagulation and other clinical treatments may affect the prognoses of patients with PVT and how to balance the risks and benefits of treatment remain unresolved [10].

Previous studies have shown that the prognosis of PVT in patients with cirrhosis is associated with the severity of thromboembolism and liver disease [2, 11]. Furthermore, the risk factors for GIB in patients with PVT include esophageal varices, superior mesenteric vein (SMV) thrombosis, and ascites [1]. However, these studies only focused on the risk factors for PVT. Additionally, laboratory analysis results or clinical symptoms alone were insufficient when assessing the prognosis. Because an accurate prognostic assessment is critical for physicians and patients, it is necessary to quantify clinical variables to achieve individualized prognosis predictions for patients with PVT. In these cases, a nomogram is an intuitive presentation form for prediction models [12]. To the best of our knowledge, no study has reported the use of prediction models or nomograms for predicting the prognosis of patients with PVT. Therefore, this study aimed to develop and validate nomogram predictive models for assessing the long-term prognoses of patients with PVT to facilitate the accurate evaluation of the clinical statuses of patients and timely initiation of appropriate treatments and interventions.

## Materials and methods

### Study setting and participants

This retrospective clinical study was performed at Shanghai East Hospital, Shanghai, China and Shanghai Tongji Hospital, Shanghai, China from January 2012 to May 2021. Overall, 471 patients with PVT were enrolled; 46 patients with insufficient clinical information were excluded. The internal cohort included 334 patients from Shanghai East Hospital, and the external validation cohort consisted of 91 patients from Shanghai Tongji Hospital. Patients with insufficient clinical information were excluded from this study.

The inclusion criteria were as follows: (1) sufficient clinical information, including medical history, clinical manifestations, physical signs, and laboratory examination results; and (2) diagnosis of PVT using at least two diagnostic methods, including portal vein Doppler ultrasound, contrast-enhanced computed tomography (CT) of the abdomen, contrast-enhanced magnetic resonance imaging (MRI) of the abdomen, and portal vein angiography. The following imaging information were utilized to diagnose PVT (all with sensitivities and specificities ranging from 90 to 100%) [13]: (1) portal vein Doppler ultrasound scan showing solid isoechoic or hypoechoic material within the portal vein that either partially or completely fills the lumen and with absent or reduced portal venous flow; (2) contrast-enhanced CT or MRI scan of the abdomen showing a either a benign thrombus that is typically seen as a low-density non-enhancing defect within the portal vein or a tumor thrombus that is enhanced following administration of a contrast agent with distension of the vessel wall or intra-thrombus contrast enhancement; and (3) portal vein angiography showing either a filling defect or complete absence of contrast agent within the portal vein, and distal obstruction and dilatation, tortuosity, lengthening, and displacement of the splenic vein.

**Fig. 1:**
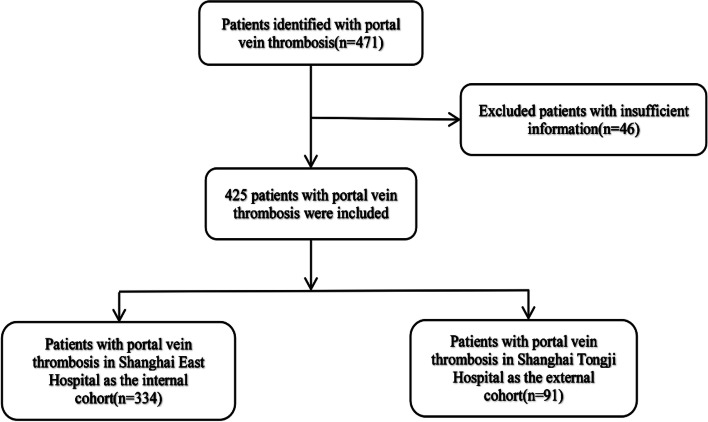
Patient selection for the two cohorts in the study

A flow chart of the screening process is shown in Fig. 1. All procedures were performed following the ethical standards of the responsible committee on human experimentation (institutional and national) and in accordance with the Helsinki Declaration of 1975 as revised in 2008. The study protocol was reviewed and approved by the Human Participants Ethics Committees of the two hospitals ([2022] Research Approval No. 012). Informed consent was submitted by all subjects when they were enrolled.

### Endpoints

The primary endpoints were GIB, PVT resolution, and death. GIB includes upper and lower GIB. Resolution or progression of PVT was determined only in patients who underwent imaging assessments more than 3 months after the date of diagnosis. PVT resolution was defined as partial thrombus resorption or complete recanalization of the PV lumen, whereas PVT progression was defined as an extension of the area of portal vein embolism or extension of the thrombus to other lumens (e.g., splenic and superior mesenteric veins). Death events were defined as all-cause mortality events.

### Data collection

Clinical data, which were recorded from the date of diagnosis to either the date of occurrence of the different clinical endpoints, the end of the study (July 16, 2021), or the date of the last follow-up visit, were collected. The baseline characteristics and pre-treatment endpoint data of the patients, including demographic information, body mass index, comorbidities, history of smoking, alcohol consumption status, GIB, concomitant use of medications, history of abdominal surgery and infection, laboratory and endoscopic characteristics, imaging features, treatment protocol with or without anticoagulation therapy, endoscope ligation, splenectomy, or abdominal surgery, were obtained. The occurrence of any of the three clinical endpoints was retrospectively recorded before the last visit.

### Statistical analysis

#### Univariate analysis

Continuous variables were expressed as means ± standard deviation or as median (interquartile range) based on the data distribution, and categorical data were presented as frequencies (percentages). The Cox regression model was used to conduct univariate analysis to identify potential predictors of the outcomes of PVT.

#### Development and validation of the prognostic model

Variables with *P-*values < 0.1 were included in the multivariate analysis. A multivariate Cox regression analysis was performed to develop the final prediction models. Calibration plots were generated to assess the similarity between the predicted and actual outcomes. Nomograms were used to visualize the prediction models. The concordance index (C-index) was calculated to evaluate the predictive ability of each model; a higher C-index indicated better discrimination. All statistical analyses were performed using SAS version 9.4 (SAS Institute Inc, Cary, NC, USA). The nomogram and calibration plots were constructed using R software version 3.6.0 (http://cran.r-project.org).

## Results

### Demographic and clinical characteristics of patients in the internal cohort

A total of 334 patients with PVT (*n* = 220; men, 65.9%), with an average age of 60.8 ± 11.6 years, were enrolled in the internal cohort. We retrieved all patients’ medical records during hospitalization, which ranged from 3 days to 110.3 months. The median follow-up time was 4.2 months (interquartile range: 15.92). The demographic and clinical characteristics of patients are summarized in Table 1. The main etiologies of the patients were hepatocellular carcinoma (44.9%), cirrhosis (44.6%), extrahepatic malignancies (20.7%), and acute intra-abdominal inflammatory disease (7.2%). Thrombolytic therapy was administered to 85 (85/334, 25.4%) patients; anticoagulants included low-molecular-weight heparin (*n* = 32; 32/85, 37.6%), warfarin (*n* = 18; 18/85, 21.2%), and rivaroxaban (*n* = 7; 7/85, 8.2%); antiplatelets included aspirin (*n* = 26; 26/85, 30.6%) and clopidogrel hydrogen sulfate (*n* = 2; 2/85, 2.4%). The mean duration of thrombolytic treatment was 2.4 months (range, 3 days–19.9 months). Among the 57 patients who received anticoagulation therapy, 5 (5/57, 8.8%) had GIB and 9 (9/57, 15.8%) died. Forty-nine of these 57 patients were assessed for resolution or progression of PVT: 4 (4/49, 8.2%) showed PVT progression and 31 (31/49, 63.3%) showed PVT resolution. Meanwhile, of the 28 patients who received antiplatelet therapy, 4 (4/28, 14.3%) had GIB, 8 (8/28, 25.6%) died, and 22 (22/28, 78.6%) were assessed for resolution or progression of PVT: 4 (18.2%) showed PVT progression and 10 (45.5%) showed PVT resolution. Among the 249 patients who did not receive thrombolytic therapy, 24 (24/249, 9.6%) had GIB, 35 (35/249, 14.1%) died, and 116 (116/249, 46.6%) were assessed for resolution or progression of PVT; of those who were further assessed, 31 (31/116, 26.7%) showed PVT progression and 44 (44/116, 37.9%) showed PVT resolution. Anticoagulation therapy significantly accelerated PVT resolution (hazard ratio [HR] = 2.81, 95% confidence interval [CI] = 1.73–4.56, *P* < 0.0001). Additionally, anticoagulation therapy did not lead to GIB or death (*P* > 0.1). The types of antiplatelets used was not included in the final statistical analysis because of limited data availability.

**Table 1:** Demographic and clinical characteristics of patients in the internal cohort

Variable	
**Age (yr)**	60.79 ± 11.57
**Sex, n (%)**	
female	114(34.13)
male	220(65.87)
**BMI(kg/m**^**2**^**)**	22.73 ± 3.36
**Drinking, n (%)**	
No	220(65.87)
Yes	114(34.13)
**Smoking, n (%)**	
No	239(71.56)
Yes	95(28.44)
**Comorbidities**	
Liver disease, n (%)	
No	35(10.48)
Cirrhosis	149(44.61)
Hepatic carcinoma	150(44.91)
Extrahepatic disease, n (%)	
No	232(69.46)
Extrahepatic malignant carcinoma	67(20.06)
Acute abdominal infection	24(7.19)
Others	11(3.29)
Current gastrointestinal bleeding, n (%)	
NO	267(79.94)
YES	67(20.06)
History of gastrointestinal bleeding, n (%)	
NO	258(77.25)
YES	76(22.75)
History of abdominal infection, n (%)	
NO	164(49.10)
YES	170(50.90)
History of anticoagulants, n (%)	
NO	304(91.02)
YES	30(8.98)
History of hepatic encephalopathy, n (%)	
NO	326(97.60)
YES	8(2.40)
History of abdominal infection, n (%)	
NO	154(46.11)
YES	180(53.89)
History of blood transfusion, n (%)	
NO	250(74.85)
YES	84(25.15)
History of anti-hypertensive drugs, n (%)	
NO	251(75.15)
YES	13(3.89)
Atrial fibrillation, n (%)	
NO	316(94.61)
YES	18(5.39)
Coronary heart disease, n (%)	
NO	309(92.51)
YES	25(7.49)
Cerebral infarction, n (%)	
NO	305(91.32)
YES	29(8.68)
Diabetes, n (%)	
NO	240(71.86)
YES	94(28.14)
**The site of thrombus involvement**	
Main portal vein, n (%)	
NO	86(25.75)
YES	248(74.25)
Portal branch, n (%)	
NO	126(37.72)
YES	208(62.28)
Mesenteric vein, n (%)	
NO	252(75.45)
YES	82(24.55)
Splenic vein, n (%)	
NO	283(84.73)
YES	51(15.27)
Others, n (%)	
NO	299(89.52)
YES	35(10.48)
**Esophageal varices, n (%)**	
NO	133(39.82)
YES	201(60.18)
**Degree of esophageal varices, n (%)**	
mild	24(7.19)
moderate	74(22.16)
severe	103(30.84)
**Red color sign, n (%)**	
NO	274(82.04)
YES	60(17.96)
**History of endoscopic operation, n (%)**	
NO	283(84.73)
YES	51(15.27)
**Ascites, n (%)**	
NO	161(48.20)
YES	173(51.80)
**Splenomegaly, n (%)**	
NO	153(45.81)
YES	181(54.19)
**CTP classification, n (%)**	
Level A	172(51.50)
Level B	135(40.42)
Level C	27(8.08)
**Laboratory findings**	
PT(s), n (%)	
≤ 12.1	43(12.87)
> 12.1	291(87.13)
INR, n (%)	
0.8–1.5	308(92.22)
> 1.5	26(7.78)
D-dimer(mg/L), n (%)	
≤ 0.55	25(7.49)
> 0.55	309(92.51)
WBC(× 10^9^/L), n (%)	
3.5–9.5	191(57.19)
< 3.5	85(25.45)
> 9.5	58(17.37)
NEUT(× 10^9^/L), n (%)	
1.8–6.3	125(37.43)
< 1.8	176(52.69)
> 6.3	33(9.88)
LYMPH(× 10^9^/L), n (%)	
1.1–3.2	115(34.43)
< 1.1	215(64.37)
> 3.2	4(1.20)
RBC(× 10^12^/L), n (%)	
< 3.8	176(52.69)
≥ 3.8	158(47.31)
HB(g/L), n (%)	
< 115	194(58.08)
≥ 115	140(41.92)
HCT(%), n (%)	
< 35	195(58.38)
≥ 35	139(41.62)
PLT(× 10^9^/L), n (%)	
< 125	155(46.41)
≥ 125	179(53.59)
CRP(mg/L), n (%)	
≤ 5	88(26.35)
> 5	246(73.65)
ALB(g/L), n (%)	
< 40	262(78.44)
≥ 40	72(21.56)
ALT(U/L), n (%)	
≤ 40	226(67.66)
> 40	108(32.34)
AST(U/L), n (%)	
≤ 35	146(43.71)
> 35	188(56.29)
TBIL(μmol/L), n (%)	
≤ 21	183(54.79)
> 21	151(45.21)
DBIL(μmol/L), n (%)	
≤ 10.2	167(50.00)
> 10.2	167(50.00)
GGT(U/L), n (%)	
≤ 45	111(33.23)
> 45	223(66.77)
ALP(U/L), n (%)	
≤ 100	151(45.21)
> 100	183(54.79)
LDH(U/L), n (%)	
≤ 250	187(55.99)
> 250	147(44.01)
SCR(μmol/L), n (%)	
≤ 92	277(82.93)
> 92	57(17.07)
BUN(ng/mL), n (%)	
≤ 6.1	220(65.87)
> 6.1	114(34.13)
UA(μmol/L), n (%)	
≤ 369	258(77.25)
> 369	76(22.75)
TC(mmol/L), n (%)	
≤ 6.22	328(98.20)
> 6.22	6(1.80)
TG(mmol/L), n (%)	
≤ 2.26	302(90.42)
> 2.26	32(9.58)
HDL(mmol/L), n (%)	
< 1.15	236(70.66)
≥ 1.15	98(29.34)
LDL(mmol/L), n (%)	
≤ 4.14	184(55.09)
> 4.14	150(44.91)
K(mmol/L), n (%)	
3.5–5.1	258(77.25)
< 3.5	68(20.36)
> 5.1	8(2.40)
Na(mmol/L), n (%)	
137–145	214(64.07)
< 137	109(32.63)
> 145	11(3.29)
AFP(mmol/L), n (%)	
≤ 7	230(68.86)
> 7	104(31.14)
**Treatment and symptoms after diagnosis of PVT**	
Hepatic encephalopathy, n (%)	
NO	309(92.51)
YES	25(7.49)
Endoscope ligation, n (%)	
NO	323(96.71)
YES	11(3.29)
Splenectomy, n (%)	
NO	305(91.32)
YES	29(8.68)
Abdominal infection, n (%)	
NO	302(90.42)
YES	32(9.58)
Blood transfusion, n (%)	
NO	266(79.64)
YES	68(20.36)
Abdominal surgery, n (%)	
NO	259(77.54)
YES	75(22.46)
Thrombolytic therapy, n (%)	
NO	249(74.55)
anticoagulants	57(17.07)
anticoagulants	28(8.38)
**The drugs of thrombolytic therapy, n (%)**	
Anticoagulant drugs	
low molecular weight heparin	32(37.65)
warfarin	18(21.18)
rivaroxaban	7(8.24)
Antiplatelet drugs	
aspirin	26(30.59)
clopidogrel hydrogen sulfate	2(2.34)

*BMI* Body mass index, *PT* Prothrombin time, *INR* International normalized ratio, *WBC* White blood cell, *RBC* Red blood cell, *HB* Hemoglobin, *PLT* Platelet count, *CRP* C-reactive protein, *ALB* Albumin, *ALT* Alanine aminotransferase, *AST* Aspartate aminotransferase, *TBIL* Total bilirubin, *DBIL* Direct bilirubin, *GGT* Gamma-glutamyltransferase*, SCr* Serum creatinine, *LDH* Lactate dehydrogenase, *TG* Triglyceride, *HDL* High-density lipoprotein, *LDL* Low-density lipoprotein, *K* Kalium, *AFP* Alpha-fetoprotein

Overall, 33 (9.9%, 33/334) GIB events were recorded in the internal cohort. The main cause of GIB was esophagogastric variceal bleeding (n = 30; 90.9%); only three (9.1%) cases were attributable to lower GIB. The median duration between the diagnosis of PVT and occurrence of GIB events was 7.4 months (range, 3 days–49.8 months; interquartile range: 12.9). The mortality rate of GIB was 24.2% (8/33). Patients with cirrhosis had the highest incidence of GIB (26/33; 78.8%) followed by those with hepatocellular carcinoma (5/33; 15.2%). Only one case of GIB was recorded among patients with abdominal infections and those with extrahepatic malignancies.

A total of 187 patients underwent assessments to determine progression or resolution of PVT. PVT progressed, resolved, and was unchanged in 39 (20.9%), 85 (45.5%), and 63 (33.7%) patients, respectively; the mortality rate of PVT progression was 2.6% (1/39). One patient who did not receive anticoagulation therapy had a fatal intestinal infarction.

A total of 52 (52/334, 15.6%) patients with an average age of 65.2 ± 12.2 years (range, 44–88 years) died in this cohort. The median overall survival was 6.6 months (range, 5 days–67.7 months; interquartile range: 15.18). The main causes of death were related to the primary disease. Patients with malignant tumors had the highest mortality rate (25/52; 48.1%) followed by patients with GIB (12/52; 23.1%).

### Development of the prognostic nomogram

**Fig. 2:**
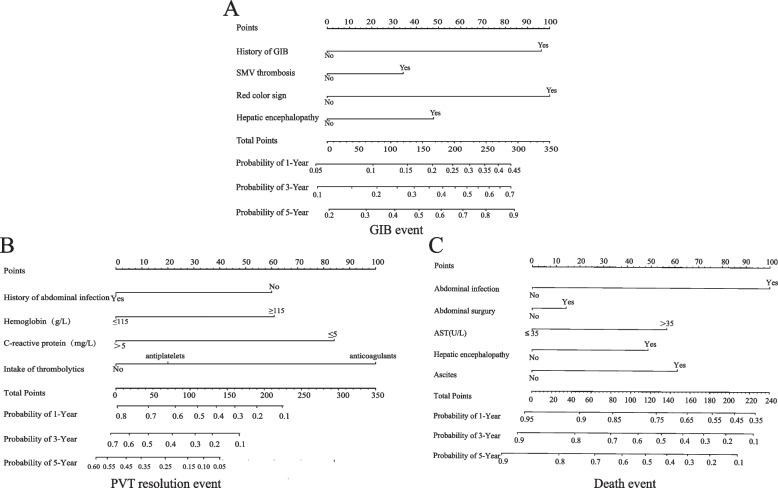
Nomogram plots of three clinical outcomes in patients with PVT. **A** Nomogram plot of gastrointestinal bleeding events. **B** Nomogram plot of portal vein thrombus resolution events. **C** Nomogram plot of death event

The univariate predictors of PVT resolution events are shown in Table 2, whereas the univariate predictors of other clinical endpoints are outlined in Supplementary Tables 1 and 2. After purposefully removing non-significant variables from the multivariate analysis, we identified predictors for the three clinical endpoints (Table 3). Four predictors of GIB events were identified: a history of GIB (HR = 2.16, 95% CI = 0.96–4.88), SMV thrombosis (HR = 1.31, 95% CI = 0.63–2.73), red color sign observed during endoscopy (HR = 2.22, 95% CI = 1.01–4.90), and hepatic encephalopathy (HR = 1.46, 95% CI = 0.6–3.55). Additionally, five predictors of PVT resolution were identified: history of abdominal infection (HR = 0.54, 95% CI = 0.35–0.85), high hemoglobin levels (HR = 1.88, 95% CI = 1.20–2.96), high C-reactive protein levels (HR = 0.42, 95% CI = 0.27–0.67), antiplatelet therapy (HR = 1.22, 95% CI = 0.60–2.48), and anticoagulant therapy (HR = 2.81, 95% CI = 1.73–4.56). Furthermore, five predictors of death were identified: abdominal infection (HR = 1.89, 95% CI = 1.05–3.4), abdominal surgery (HR = 1.5, 95% CI = 0.83–2.69), aspartate aminotransferase level (HR = 2.23, 95% CI = 1.26–3.96), hepatic encephalopathy (HR = 0.78, 95% CI = 0.19–3.27), and ascites (HR = 1.95, 95% CI = 1.09–3.51). The final Cox regression models were presented as nomograms incorporating the aforementioned independent predictors (Fig. 2). The model was applied as follows: the total scores were obtained by calculating the sum of the scores of the predictors, and the scores were used to predict the incidence of the three clinical endpoints at 1, 3, and 5 years, or at 1, 2, and 3 years.

**Table 2:** Univariate analysis of portal vein thrombus resolution events

Variable	Coefficient	Stat	HR (95%CI)	*P* value
Age	0.02	2.86	1.02(1.00,1.04)	0.0909
BMI	0.01	0.01	1.00(0.94,1.08)	0.9029
Drinking	-0.35	2.10	0.70(0.44,1.13)	0.1473
Smoking	-0.48	3.54	0.62(0.38,1.02)	0.0599
**Liver disease**				
Cirrhosis	-0.59	3.46	0.55(0.30,1.03)	0.0628
Hepatic carcinoma	-0.32	0.81	0.73(0.37,1.45)	0.3684
**Comorbidities**				
Atrial fibrillation	0.67	2.04	1.95(0.78,4.88)	0.1530
Coronary heart disease	0.41	1.29	1.50(0.75,3.02)	0.2562
Cerebral infarction	0.22	0.40	1.24(0.64,2.41)	0.5257
Diabetes	-0.19	0.61	0.83(0.52,1.33)	0.4345
Ascites	-0.41	3.31	0.67(0.43,1.03)	0.0687
Splenomegaly	-0.34	2.37	0.71(0.47,1.10)	0.1235
Current GIB	-0.13	0.26	0.88(0.54,1.44)	0.6126
History of GIB	-0.34	1.82	0.71(0.44,1.17)	0.1776
History of abdominal surgery	0.35	2.39	1.42(0.91,2.23)	0.1218
History of anticoagulants	0.63	3.59	1.87(0.98,3.57)	0.0580
History of blood transfusion	-0.11	0.21	0.90(0.56,1.44)	0.6501
History of anti-hypertensive drugs	-0.29	0.39	0.75(0.30,1.86)	0.5334
History of endoscopic operation	0.44	1.81	1.56(0.82,2.98)	0.1781
History of hepatic encephalopathy	-0.48	0.65	0.62(0.20,1.97)	0.4197
History of abdominal infection	-0.51	5.23	0.60(0.39,0.93)	0.0222
**The site of thrombus involvement**				
Main portal vein thrombosis	-0.13	0.26	0.88(0.54,1.45)	0.6112
Portal branch thrombosis	-0.10	0.20	0.91(0.59,1.39)	0.6514
SMV thrombosis	-0.28	1.38	0.76(0.48,1.21)	0.2405
Splenic vein thrombosis	0.17	0.39	1.18(0.70,1.99)	0.5301
**Esophageal varices**	-0.44	3.38	0.64(0.40,1.03)	0.0660
Red color sign	-0.44	2.53	0.64(0.37,1.11)	0.1117
**Degree of esophageal varices**				
mild	REF			
moderate	0.20	0.20	1.22(0.51,2.94)	0.6526
severe	0.23	0.29	1.26(0.55,2.88)	0.5896
**CTP classification**				
Level A	REF			
Level B	-0.50	4.47	0.61(0.38,0.96)	0.0345
Level C	-0.23	0.24	0.79(0.31,2.02)	0.6276
**Laboratory findings**				
PT > 12.1(s)	-0.20	0.35	0.82(0.42,1.60)	0.5554
INR > 1.5	0.04	0.01	1.05(0.45,2.45)	0.9174
D-dimer > 0.55(mg/L)	0.03	0.01	1.03(0.41,2.56)	0.9490
WBC (× 10^9^/L)				
< 3.5	-0.19	0.54	0.83(0.51,1.36)	0.4616
> 9.5	-0.07	0.05	0.93(0.52,1.68)	0.8172
RBC ≥ 3.8 (× 10^12^/L)	0.41	3.26	1.51(0.97,2.37)	0.0711
HB ≥ 115(g/L)	0.49	4.68	1.64(1.05,2.56)	0.0306
PLT ≥ 125 (× 10^9^/L)	0.21	0.90	1.23(0.80,1.89)	0.3421
CRP > 5(mg/L)	-0.64	8.16	0.53(0.34,0.82)	0.0043
ALB ≥ 40(g/L)	0.65	6.39	1.91(1.16,3.15)	0.0115
ALT > 40(U/L)	-0.25	0.71	0.78(0.44,1.39)	0.3983
AST > 35(U/L)	0.41	3.40	1.51(0.97,2.34)	0.0652
TBIL > 21(μmol/L)	0.13	0.35	1.14(0.74,1.77)	0.5528
DBIL > 10.2(μmol/L)	-0.05	0.06	0.95(0.61,1.47)	0.8068
SCR > 92(μmol/L)	0.01	0.01	1.00(0.56,1.78)	0.9952
TG > 2.26(mmol/L)	-0.29	0.52	0.75(0.34,1.64)	0.4719
HDL ≥ 1.15(mmol/L)	0.10	0.20	1.11(0.71,1.73)	0.6585
LDL > 2.16(mmol/L)	0.08	0.15	1.09(0.71,1.67)	0.7023
K < 3.5(mmol/L)	0.14	0.27	1.15(0.67,1.98)	0.6043
AFP > 7(ng/mL)	0.23	0.62	1.26(0.71,2.22)	0.4307
**Treatment and symptoms after diagnosis of PVT**				
Hepatic encephalopathy	-0.64	2.84	0.53(0.25,1.11)	0.0919
Endoscope ligation	0.62	1.78	1.86(0.75,4.64)	0.1825
Abdominal infection	0.22	0.35	1.25(0.60,2.59)	0.5542
Blood transfusion	-0.26	1.01	0.77(0.46,1.29)	0.3161
Abdominal surgery	0.14	0.32	1.15(0.71,1.87)	0.5732
Thrombolytic therapy	0.55	6.34	1.73(1.13,2.66)	0.0118
**The drugs of thrombolytic therapy**				
no drugs	REF			
anticoagulants	0.80	11.00	2.22(1.39,3.55)	0.0009
antiplatelets	0.04	0.02	1.05(0.52,2.09)	0.9017
**Anticoagulant drugs**				
low molecular weight heparin	REF			
warfarin	1.56	1.83	4.78(0.50,46.07)	0.1760
**Anticoagulant duration**	0.01	0.06	1.00(1.00,1.01)	0.8009

*HR* Hazard ratio, *CI* Confidence interval, *BMI* Body mass index, *GIB* Gastrointestinal bleeding, *SMV* Superior mesenteric vein, *PT* Prothrombin time, *INR* International normalized ratio, *WBC* White blood cell, *RBC* Red blood cell, *HB* Hemoglobin, *PLT* Platelet count, *CRP* C-reactive protein, *ALB* Albumin, *ALT* Alanine aminotransferase, *AST* Aspartate aminotransferase, *TBIL* Total bilirubin, *DBIL* Direct bilirubin, *SCR* Serum creatinine, *TG* Triglyceride, *HDL* High-density lipoprotein, *LDL* Low-density lipoprotein, *K* Kalium, *AFP* Alpha-fetoprotein

**Table 3:** Multivariate analysis of three clinical outcomes of PVT in the internal cohort

Variable	Coefficient	Stat	HR (95%CI)	*P* value
**Gastrointestinal bleeding events**				
History of GIB	0.77	3.42	2.16(0.96,4.88)	0.0645
SMV thrombosis	0.27	0.53	1.31(0.63,2.73)	0.4675
Red color sign	0.80	3.92	2.22(1.01,4.90)	0.0478
Hepatic encephalopathy	0.38	0.69	1.46(0.60,3.55)	0.4068
**Portal vein thrombus resolution events**				
History of abdominal infection	-0.62	7.28	0.54(0.35,0.85)	0.0070
HB ≥ 115(g/L)	0.63	7.50	1.88(1.20,2.96)	0.0062
CRP > 5(mg/L)	-0.86	13.91	0.42(0.27,0.67)	0.0002
Anticoagulant therapy	1.03	17.31	2.81(1.73,4.56)	< 0.0001
Antiplatelet therapy	0.20	0.31	1.22(0.60,2.48)	0.5755
**Death events**				
Abdominal infection	0.64	4.48	1.89(1.05,3.41)	0.0343
Abdominal surgery	0.40	1.83	1.51(0.83,2.69)	0.1764
AST > 35 (U/L)	0.80	7.57	2.23(1.26,3.96)	0.0059
Hepatic encephalopathy	0.25	0.12	0.78(0.19,3.27)	0.7323
Ascite	0.67	5.02	1.95(1.09,3.51)	0.0251

*HR* Hazard ratio, *CI* Confidence interval, *GIB* Gastrointestinal bleeding, *SMV* Superior mesenteric vein, *HB* Hemoglobin, *AST* Aspartate aminotransferase, *CRP* C-reactive protein

### Validation and clinical use of the nomogram

During internal validation, the C-indices of GIB, PVT resolution, and mortality were 0.69, 0.73, and 0.75, respectively (Table 4). During external validation, the C-indices of the three clinical endpoints were 0.82, 0.68, and 0.78, respectively, which was mostly better than the C-indices of the internal cohort and indicated the satisfactory diagnostic power of the nomogram models.

**Table 4:** Model performance parameters (C-index)

Clinical endpoint	Internal cohort	External cohort
Gastrointestinal bleeding	0.69	0.82
Portal vein thrombus resorption	0.73	0.68
Death	0.75	0.78

*C-index* Concordance index

**Fig. 3:**
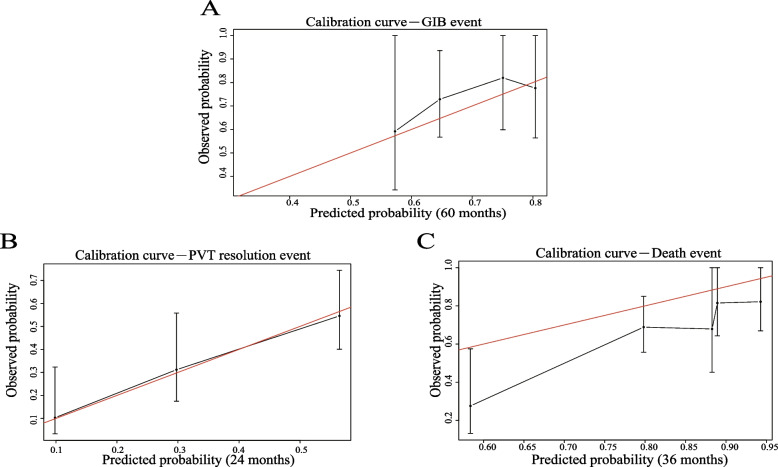
Calibration curves for three nomograms to predict prognoses in patients with PVT in the internal cohort. **A** Calibration curve of the 60-month nomogram for gastrointestinal bleeding events. **B** Calibration curve of the 24-month nomogram for PVT resolution event. **C** Calibration curve of the 36-month nomogram for death event

The results of the calibration plots for the nomograms showed consistency between the predicted and observed values (Fig. 3). The X-axis represents the occurrence of different clinical outcomes as predicted by the nomogram, whereas the Y-axis represents the actual outcomes. The red line represents the perfect prediction of an ideal model. The black line represents the performance of our nomogram model. The closer the black line is to the gray line, the better the predicted value.

The predictive power of the nomograms can be determined through practical applications. For example, regarding the calibration curve of the nomogram prediction model for GIB events, the model will underestimate the risk of GIB when the predicted probability of GIB ranges from 60 to 75%. Therefore, appropriate treatment for patients with PVT may be deferred in clinical settings. However, the prediction is remarkably close to the actual outcome during this period, which makes no difference in clinical settings. When the prediction of GIB is between 75 and 80%, the nomogram model will overestimate the risk of GIB, which indicates the need for the timely initiation of treatment for patients at high risk for bleeding. Therefore, the implementation of this nomogram model has strong clinical value.

## Discussion

We comprehensively analyzed the long-term clinical outcomes of patients with PVT, identified predictors of the three clinical outcomes, and established three simple, intuitive, and rapid prognostic prediction models. Additionally, we evaluated the effects of anticoagulation therapy on PVT. The results indicated that anticoagulation therapy may increase the rate of recanalization without increasing the risk of GIB and death.

Recently, the incidence of PVT, which is estimated to range from 1.6 to 15.8% in patients with cirrhosis or portal hypertension, has gradually increased due to improvements in diagnostics brought about by advancements in imaging modalities [4]. PVT increases the risk of portal vein hypertension and related complications, such as bleeding, thrombus progression, and death [8, 14, 15]. Therefore, monitoring the prognosis of patients with PVT is crucial for clinical decision-making. According to recent studies, the incidence rates of bleeding, PVT resolution, PVT progression, and death after a diagnosis of PVT ranges from 12 to 30.8%, 31.6 to 71%, 5.7 to 15.8%, and 13 to 24.2%, respectively [14–17]. In the present study, the overall incidence rates of GIB and death were 9.9, and 15.6%, respectively, while 45.5 and 20.9% of patients who have been evaluated for morphological change of PVT were observed to have PVT resolution and progression, respectively, which are similar to the previously reported rates.

Previous studies have confirmed that anticoagulation therapy, an interval of less than 6 months between a diagnosis of thrombosis and initiation of anticoagulation therapy, and splenic thickness may be positively associated with portal vein recanalization [7, 18, 19]. Anticoagulation therapy is a crucial treatment option for patients with PVT; however, it was rarely implemented previously as clinicians and patients were concerned about the risk of complications such as GIB [16]. Moreover, among the patients who were evaluated for morphological PVT changes in the present study, the PVT recanalization rate (45.5%) was higher than the proportion of patients who received thrombolytic therapy (38.0%), suggesting that spontaneous recanalization occurred in a small number of patients with PVT, similar to the findings of previous studies [6, 17]. Notably, portal vein hypertension is a predictor of nonresponse to anticoagulation therapy [9, 20]. In our study, 63.3% of patients treated with anticoagulation therapy achieved thrombus resolution; this result is consistent with those of previous studies (30–80%) [8, 21] and also confirmed that anticoagulation therapy was a significant predictor of PVT resolution. Therefore, we suggest that most patients with PVT should receive anticoagulation therapy unless there is a high risk of bleeding.

Hemoglobin is essential for maintaining cellular bioenergetic homeostasis and modulating cell functions (inflammation and redox status of cells) through its ability to bind and transport oxygen to tissues, which may decrease the incidence of thrombosis [22]. However, excessively high levels of hemoglobin can lead to local inflammation and even tissue damage among patients with hemoglobinemia, which further accelerates the formation of thromboses [23]. The C-reactive protein level is a common indicator of inflammation and infection and is often used to assess their severity [24]. Darzi et al*.* demonstrated that a C-reactive protein level > 10 mg/L was positively associated with venous thromboembolism and that it could lead to a transient hypercoagulable state [25]. Additionally, the 2020 Chinese consensus regarding PVT indicated that inflammation or infection of the abdominal cavity may be an important risk factor for PVT in patients with cirrhosis [3]. PVT may be a potential consequence of any inflammatory intra-abdominal process (including cholecystitis, pancreatitis, or inflammatory bowel disease), and its risk will increase in a setting of acute infection and recurrent infections [26]. Therefore, patients with a higher C-reactive protein level and a history of abdominal infection may have higher levels of inflammation, which lowers the probability of PVT resolution.

Portal vein hypertension is the main determinant of esophagogastric variceal bleeding [11]. Previous studies have demonstrated that esophageal varices, the red color sign observed during endoscopy, advanced stage of liver disease (Child–Turcotte–Pugh class C patients), and ascites were possible predictors of bleeding events [16, 27]. Although the same conclusions were reached in the present study, we also demonstrated that a history of GIB and thrombus extension into the mesenteric veins were significant predictors of GIB. Portal vein hypertension results in redistribution and increased blood flow through the short gastric and coronary veins, causing esophagogastric varices. Esophagogastric varices begin to form at a pressure gradient of 8–10 mmHg, and bleeding risk increases at a gradient of at least 12 mmHg [27]. Certain endoscopic variceal stigma, collectively referred to as “red color sign” (red-whale markings, nipple symptoms, cherry-red spots), correlated with a significantly higher risk of acute variceal bleeding and re-bleeding [28]; hence, early preventive endoscopic treatment and shortening of the prothrombin time may decrease the occurrence of GIB [3]. Additionally, when hepatic encephalopathy occurs in patients with advanced liver disease due to liver failure, and imaging also shows PVT extension into the superior mesenteric vein, further reductions in the flow velocity and increases in the portal vein pressure, even GIB, may occur. In the long-term, approximately 70% of patients with GIB may experience further variceal bleeding because of superficial varices and a thinner vessel wall [29]. Therefore, a history of GIB may increase the risk of re-bleeding. These new findings may help clinicians identify patients at high risk for GIB, and they may also facilitate the timely initiation of anticoagulation therapy.

Patients with cirrhosis or hepatocellular carcinoma are in a state of imbalanced coagulation function that can promote the propensity for bleeding or thrombosis [4, 30], thus making it challenging for clinicians to initiate anticoagulation therapy for PVT. Mohan et al. reported that the incidence of GIB for patients receiving anticoagulation therapy was 7.8% [31], which is very close to the rate (8.8%) observed in the present study. Furthermore, the univariate analysis performed in the present study showed that anticoagulation therapy did not promote GIB events, thus demonstrating the safety and efficacy of anticoagulation therapy for PVT. Therefore, our conclusion is consistent with those of existing studies. Qi et al*.* confirmed that prophylactic anticoagulation therapy for deep venous thrombosis in hospitalized patients with cirrhosis and without active bleeding was safe and did not increase the incidence of GIB or death [18]. Furthermore, Ageno et al. found that the duration of anticoagulation therapy was associated with a reduced risk of bleeding [21]. However, this variable was not statistically significant in the univariate analysis for GIB events in the present study, which may be due to its dual effect on GIB. Prolonging anticoagulation therapy will increase the risk of bleeding by preventing coagulation. Simultaneously, anticoagulation therapy may reduce the severity of esophagogastric varices due to PVT resolution, which decreases the incidence of GIB.

Regarding the analysis of the overall death of patients in our study, most of the fatal events were related to the underlying disease. In one case, the progression of PVT into the superior mesenteric vein caused intestinal obstruction, resulting in death. In a large prospective study of 178 patients with PVT, few deaths occurred during follow-up, and the 5-year survival rate was 96% [11]. Moreover, overall 5-year survival rates ranging from 70 to 78% have been reported by a previous study [10]. In the present study, 23.1% of all deaths were caused by fatal GIB events; however, GIB caused only 3% of the deaths in a study of 120 non-cirrhotic patients with PVT [32]. Most patients in our study had cirrhosis, hepatocellular carcinomas, and coagulation disorders, which led to a higher mortality rate. Significantly relevant factors for PVT-related mortality events include the Child–Turcotte–Pugh score, age, and ascites [32]. In partial agreement with the results of previous studies, the univariate analysis performed during the present study confirmed that the Child–Turcotte–Pugh score was related to survival (*P* = 0.0002). Because multiple variables of the Child–Turcotte–Pugh score may interact with each other during statistical analyses, these variables were analyzed separately in our study. We found that hepatic encephalopathy and ascites were independent predictors of PVT progression in the Cox regression analysis of death. Both hepatic encephalopathy and ascites are important signs of liver failure, which has a high risk for mortality and allows for a more accurate assessment of a patient’s prognosis. In addition, abdominal infections, history of abdominal surgery, and aspartate aminotransferase level > 35 U/L were positively associated with death in the present study. Intra-abdominal infections and surgery pose serious clinical challenges, and may result in wide variety of conditions ranging from uncomplicated cases to fulminant septic shock and multi-organ dysfunction, further increasing the risk of death [26].

This study had some potential limitations. First, the effectiveness of the data analysis was limited because of the retrospective nature of the study. Additionally, the etiology ratio of PVT was not compared with those reported by other studies to confirm the external implementation performance of the nomograms. Second, some data related to PVT (e.g., portal vein velocity and morphological changes in the thrombus) were not included in this study. Moreover, many patients underwent only one blood or endoscopic examination. Therefore, a dynamic follow-up could not be performed. Future prospective, multicenter, randomized clinical trials with larger sample sizes are needed to corroborate the findings of this study.

In conclusion, we developed three easy-to-use nomogram prediction models to evaluate the prognosis and assist with the initiation of early intervention for patients with PVT. Additionally, the results of this study suggested that most patients with PVT should undergo anticoagulation therapy. Furthermore, these findings provided evidence of the benefits and risks of anticoagulation therapy for patients with PVT, which will help clinicians balance the benefit-to-risk ratio of anticoagulation therapy.

## Supplementary Information

**Additional file 1:** **Supplementary** **Table1.** Univariate analysis of gastrointestinalbleeding events. **Supplementary** **Table2. **Univariate analysis of death events. 

## Data Availability

The data that support the findings of this study are available from the corresponding author upon reasonable request.

## Abbreviations

PVT: Portal vein thrombosis
GIB: Gastrointestinal bleeding
SMV: Superior mesenteric vein
CT: Computed tomography
MRI: Magnetic resonance imaging
C-index: Concordance index
HR: Hazard ratio
CI: Confidence interval
