# Supplementary material for: Development and external validation of prognostic scoring models for portal vein thrombosis: a multicenter retrospective study
Source: Thromb J. 2023 Jan 23;21:9. doi: 10.1186/s12959-023-00455-w (PMC9869608; doi:10.1186/s12959-023-00455-w)
Supplement: Supplementary file 1 — Additional file 1: Supplementary Table1. Univariate analysis of gastrointestinalbleeding events. Supplementary Table2. Univariate analysis of death events. [file 12959_2023_455_MOESM1_ESM.doc]

Supplementary Table 1. Univariate analysis of gastrointestinal bleeding events

| **Variable** | **Coefficient** | | **Stat** | **HR (95%CI)** | ***P* value** |  |  |  |
| --- | --- | --- | --- | --- | --- | --- | --- | --- |
| Age | | 0.01 | 0.02 | 1.00(0.97,1.03) | 0.9006 |  |  |  |
| BMI | | -0.03 | 0.20 | 0.97(0.87,1.09) | 0.6527 |  |  |  |
| Drinking | | -0.04 | 0.01 | 0.96(0.47,1.98) | 0.9142 |  |  |  |
| Smoking | | 0.58 | 2.71 | 1.79(0.90,3.57) | 0.1001 |  |  |  |
| **Extrahepatic disease** | |  |  |  |  |  |  |  |
| Extrahepatic malignant carcinoma | | 2.12 | 4.35 | 0.12(0.02,0.88) | 0.0371 |  |  |  |
| Acute abdominal infection | | 1.35 | 1.75 | 0.26(0.04,1.91) | 0.1858 |  |  |  |
| **Comorbidities** | |  |  |  |  |  |  |  |
| Atrial fibrillation | | 0.18 | 0.06 | 1.20(0.29,5.00) | 0.8064 |  |  |  |
| Coronary heart disease | | -1.21 | 1.41 | 0.30(0.04,2.19) | 0.2346 |  |  |  |
| Cerebral infarction | | 0.61 | 1.60 | 1.85(0.71,4.79) | 0.2061 |  |  |  |
| Diabetes | | 0.64 | 3.32 | 1.89(0.95,3.76) | 0.0683 |  |  |  |
| Ascites | | 0.74 | 4.02 | 2.10(1.02,4.33) | 0.0451 |  |  |  |
| Splenomegaly | | 0.32 | 0.80 | 1.38(0.68,2.81) | 0.3710 |  |  |  |
| Current GIB | | 1.17 | 11.14 | 3.21(1.62,6.36) | 0.0008 |  |  |  |
| History of GIB | | 1.25 | 12.55 | 3.49(1.75,6.97) | 0.0004 |  |  |  |
| History of abdominal surgery | | -0.09 | 0.07 | 0.91(0.46,1.81) | 0.7957 |  |  |  |
| History of anticoagulant | | 0.31 | 0.33 | 1.36(0.48,3.86) | 0.5673 |  |  |  |
| History of blood transfusion | | 1.07 | 9.39 | 2.92(1.47,5.79) | 0.0022 |  |  |  |
| History of anti-hypertensive drugs | | 1.17 | 5.45 | 3.21(1.21,8.55) | 0.0195 |  |  |  |
| History of endoscopic operation | | 0.61 | 1.26 | 1.84(0.63,5.36) | 0.2626 |  |  |  |
| History of Hepatic encephalopathy | | 0.43 | 0.35 | 1.54(0.37,6.49) | 0.5543 |  |  |  |
| **The site of thrombus involvement** | |  |  |  |  |  |  |  |
| Main portal vein thrombosis | | 0.27 | 0.36 | 1.31(0.54,3.19) | 0.5481 |  |  |  |
| Portal branch thrombosis | | 0.11 | 0.10 | 1.12(0.56,2.25) | 0.7557 |  |  |  |
| SMV thrombosis | | 0.67 | 3.69 | 1.96(0.99,3.90) | 0.0548 |  |  |  |
| Splenic vein thrombosis | | 0.41 | 1.08 | 1.51(0.70,3.26) | 0.2981 |  |  |  |
| **Esophageal varices** | | 1.53 | 6.38 | 4.63(1.41,15.19) | 0.0116 |  |  |  |
| Red color sign | | 1.26 | 13.01 | 3.52(1.78,6.99) | 0.0003 |  |  |  |
| **Degree of esophageal varices**  mild | | REF |  |  |  |  |  |  |
| moderate | | -0.91 | 1.23 | 0.40(0.08,2.01) | 0.2682 |  |  |  |
| severe | | 0.81 | 1.72 | 2.24(0.67,7.49) | 0.1900 |  |  |  |
| **CTP classification** | |  |  |  |  |  |  |  |
| Level A | | REF |  |  |  |  |  |  |
| Level B | | 0.35 | 0.88 | 1.42(0.68,2.96) | 0.3486 |  |  |  |
| Level C | | 1.08 | 3.52 | 2.95(0.95,9.14) | 0.0605 |  |  |  |
| **Laboratory findings**  PT＞12.1（s） | | -0.39 | 0.62 | 0.68(0.26,1.78) | 0.4304 |  |  |  |
| INR＞1.5 | | 0.58 | 1.18 | 1.79(0.63,5.11) | 0.2770 |  |  |  |
| D-dimer＞0.55（mg/L） | | -0.48 | 0.80 | 0.62(0.22,1.77) | 0.3713 |  |  |  |
| WBC（×10^9^/L）  ＜3.5 | | 0.23 | 0.35 | 1.26(0.59,2.71) | 0.5549 |  |  |  |
| ＞9.5 | | 0.08 | 0.03 | 1.09(0.42,2.82) | 0.8618 |  |  |  |
| RBC≥3.8（×10^12^/L） | | -0.98 | 6.22 | 0.38(0.18,0.81) | 0.0127 |  |  |  |
| HB≥115（g/L） | | -0.53 | 1.96 | 0.59(0.28,1.24) | 0.1613 |  |  |  |
| PLT≥125（×10^9^/L） | | -0.62 | 2.92 | 0.54(0.26,1.10) | 0.0876 |  |  |  |
| CRP＞5（mg/L） | | -0.29 | 0.62 | 0.75(0.37,1.53) | 0.4295 |  |  |  |
| ALB≥40（g/L） | | -0.80 | 1.72 | 0.45(0.14,1.48) | 0.1894 |  |  |  |
| ALT＞40（U/L） | | 0.04 | 0.01 | 1.05(0.47,2.33) | 0.9130 |  |  |  |
| AST＞35（U/L） | | 0.16 | 0.20 | 1.17(0.59,2.33) | 0.6563 |  |  |  |
| TBIL＞21（μmol/L） | | -0.55 | 1.99 | 0.58(0.27,1.24) | 0.1586 |  |  |  |
| DBIL＞10.2（μmol/L） | | 0.31 | 0.79 | 1.37(0.69,2.71) | 0.3742 |  |  |  |
| GGT＞45（U/L） | | -0.33 | 0.89 | 0.72(0.36,1.43) | 0.3453 |  |  |  |
| SCR＞92（μmol/L） | | -0.94 | 1.66 | 0.39(0.09,1.63) | 0.1973 |  |  |  |
| LDH＞250（U/L） | | -0.98 | 5.27 | 0.38(0.16,0.87) | 0.0217 |  |  |  |
| TG＞2.26（mmol/L） | | -0.72 | 0.97 | 0.49(0.12,2.04) | 0.3248 |  |  |  |
| HDL≥1.15（mmol/L） | | 0.08 | 0.04 | 1.08(0.53,2.20) | 0.8352 |  |  |  |
| LDL＞2.16（mmol/L） | | -0.67 | 3.12 | 0.51(0.24,1.08) | 0.0774 |  |  |  |
| K＜3.5（mmol/L） | | 0.01 | 0.01 | 1.00(0.41,2.44) | 0.9987 |  |  |  |
| AFP＞7（ng/mL） | | 0.43 | 1.20 | 1.54(0.71,3.35) | 0.2729 |  |  |  |
| **Treatment and symptoms after diagnosis of PVT** | | | | | |  |  |  |
| Hepatic encephalopathy | | 0.91 | 4.45 | 2.47(1.07,5.73) | 0.0349 |  |  |  |
| Endoscope ligation | | 1.10 | 3.10 | 2.99(0.88,10.14) | 0.0784 |  |  |  |
| Splenectomy | | 0.77 | 3.28 | 2.16(0.94,4.99) | 0.0703 |  |  |  |
| Abdominal infection | | -1.15 | 1.28 | 0.32(0.04,2.32) | 0.2572 |  |  |  |
| Blood transfusion | | 1.15 | 10.78 | 3.15(1.59,6.24) | 0.0010 |  |  |  |
| Abdominal surgery | | -0.50 | 1.07 | 0.61(0.23,1.57) | 0.3012 |  |  |  |
| Thrombolytic therapy | | -0.26 | 0.43 | 0.77(0.36,1.67) | 0.5106 |  |  |  |
| **The drugs of thrombolytic therapy** | |  |  |  |  |  |  |  |
| no drugs | | REF |  |  |  |  |  |  |
| anticoagulants | | -0.27 | 0.30 | 0.76(0.29，2.01) | 0.5811 |  |  |  |
| antiplatelets | | -0.24 | 0.19 | 0.79(0.27，2.28) | 0.6594 |  |  |  |
| **Anticoagulant drugs**  low molecular weight heparin | | REF |  |  |  |  |  |  |
| warfarin | | -0.04 | 0.01 | 0.96(0.16,5.76) | 0.9636 |  |  |  |
| **Anticoagulant duration（month）**  ≤1 | | REF |  |  |  |  |  |  |
| 1-3 | | -0.76 | 1.54 | 0.47(0.14,1.55) | 0.2144 |  |  |  |
| ≥3 | | -0.42 | 0.33 | 0.66(0.16,2.76) | 0.5641 |  |  |  |

HR, hazard ratio; CI, confidence interval; BMI, body mass index; GIB, gastrointestinal bleeding; SMV, superior mesenteric vein; PT, prothrombin time; INR, international normalized ratio; WBC, White blood cell; RBC, red blood cell; HB, hemoglobin; PLT, platelet count; CRP, C-reactive protein; ALB, albumin; ALT, alanine aminotransferase; AST, aspartate aminotransferase; TBIL, total bilirubin; DBIL, direct bilirubin; GGT, gamma-glutamyltransferase; SCr, serum creatinine; LDH, lactate dehydrogenase; TG, triglyceride; HDL, high-density lipoprotein; LDL, low-density lipoprotein; K, [kalium](javascript:;); AFP, alpha-fetoprotein.

| **Supplementary Table 2. Univariate analysis of death events**   | **Variable** | **Coefficient** | **Stat** | **HR (95%CI)** | ***P* value** | | --- | --- | --- | --- | --- | | Age | 0.03 | 6.42 | 1.03(1.01,1.05) | 0.0113 | | BMI | -0.04 | 0.73 | 0.96(0.88,1.05) | 0.3940 | | Drinking | 0.20 | 0.49 | 1.22(0.70,2.14) | 0.4834 | | Smoking | -0.15 | 0.24 | 0.86(0.46,1.59) | 0.6233 | | **Liver disease** |  |  |  |  | | Cirrhosis | 1.02 | 1.95 | 2.78(0.66,11.65) | 0.1625 | | Hepatic carcinoma | 1.44 | 3.79 | 4.23(0.99,18.09) | 0.0516 | | **Comorbidities** |  |  |  |  | | Atrial fibrillation | 0.18 | 0.09 | 1.20(0.37,3.85) | 0.7611 | | Coronary heart disease | -0.47 | 0.62 | 0.63(0.20,2.01) | 0.4324 | | Cerebral infarction | -0.14 | 0.07 | 0.87(0.31,2.43) | 0.7954 | | Diabetes | 0.15 | 0.26 | 1.16(0.66,2.05) | 0.6115 | | Ascites | 0.73 | 6.13 | 2.08(1.17,3.71) | 0.0133 | | Splenomegaly | 0.14 | 0.25 | 1.15(0.66,2.01) | 0.6154 | | Current GIB | 0.32 | 1.10 | 1.37(0.76,2.47) | 0.2952 | | History of GIB | 0.09 | 0.09 | 1.10(0.61,1.98) | 0.7636 | | History of abdominal surgery | 0.57 | 4.08 | 0.57(0.32,0.98) | 0.0435 | | History of anticoagulant | -0.15 | 0.09 | 0.86(0.31,2.38) | 0.7679 | | History of blood transfusion | 0.36 | 1.53 | 1.43(0.81,2.52) | 0.2167 | | History of endoscopic operation | -0.92 | 1.61 | 0.40(0.10,1.65) | 0.2052 | | History of abdominal infection | 0.62 | 4.37 | 1.86(1.04,3.33) | 0.0366 | | **The site of thrombus involvement** | | | | | | Main portal vein thrombosis | 0.35 | 0.91 | 1.42(0.69,2.92) | 0.3394 | | Portal branch thrombosis | 0.11 | 0.15 | 1.12(0.64,1.96) | 0.6979 | | SMV thrombosis | 0.29 | 0.79 | 0.75(0.40,1.41) | 0.3733 | | Splenic vein thrombosis | 0.84 | 3.18 | 0.43(0.17,1.09) | 0.0745 | | **Esophageal varices** | -0.12 | 0.15 | 0.89(0.50,1.59) | 0.6941 | | Red color sign | 0.12 | 0.15 | 1.13(0.60,2.13) | 0.6976 | | **Degree of esophageal varices**  mild | REF |  |  |  | | moderate | -0.18 | 0.10 | 0.84(0.29,2.46) | 0.7467 | | severe | 0.01 | 0.01 | 1.01(0.38,2.71) | 0.9779 | | **CTP classification**  Level A | REF |  |  |  | | Level B | 0.50 | 2.60 | 1.65(0.90,3.02) | 0.1070 | | Level C | 1.52 | 13.55 | 4.56(2.03,10.21) | 0.0002 | | **Laboratory findings** | | | | | | PT＞12.1（s） | -0.07 | 0.03 | 0.93(0.39,2.19) | 0.8648 | | INR＞1.5 | 0.29 | 0.39 | 1.34(0.53,3.38) | 0.5324 | | D-dimer＞0.55（mg/L） | 0.13 | 0.06 | 1.14(0.41,3.16) | 0.8035 | | WBC（×109/L）  ＜3.5 | -0.50 | 2.15 | 0.61(0.31,1.18) | 0.1424 | | ＞9.5 | -0.33 | 0.71 | 0.72(0.33,1.56) | 0.4002 | | RBC≥3.8（×1012/L） | -0.38 | 1.75 | 0.69(0.39,1.20) | 0.1856 | | HB≥115（g/L） | -0.34 | 1.33 | 0.71(0.40,1.27) | 0.2487 | | HCT≥35（%） | -0.54 | 3.15 | 0.58(0.32,1.06) | 0.0757 | | PLT≥125（×109/L） | -0.50 | 3.04 | 0.61(0.35,1.07) | 0.0815 | | CRP＞5（mg/L） | 0.53 | 2.47 | 1.71(0.88,3.32) | 0.1164 | | ALB≥40（g/L） | -0.52 | 1.41 | 0.60(0.25,1.40) | 0.2347 | | ALT＞40（U/L） | 0.34 | 1.23 | 1.40(0.77,2.53) | 0.2670 | | AST＞35（U/L） | 0.74 | 6.50 | 2.09(1.19,3.70) | 0.0108 | | TBIL＞21（μmol/L） | 0.69 | 6.02 | 1.99(1.15,3.45) | 0.0141 | | DBIL＞10.2（μmol/L） | 0.86 | 8.87 | 2.35(1.34,4.13) | 0.0029 | | GGT＞45（U/L） | 0.56 | 3.35 | 1.76(0.96,3.20) | 0.0673 | | SCR＞92（μmol/L） | 0.14 | 0.14 | 1.16(0.54,2.46) | 0.7078 | | BUN＞6.1（mmol/L） | 0.08 | 0.08 | 1.08(0.61,1.92) | 0.7824 | | TC＞6.22（mmol/L） | 1.34 | 3.44 | 3.83(0.93,15.82) | 0.0637 | | TG＞2.26（mmol/L） | -0.43 | 0.67 | 0.65(0.23,1.82) | 0.4128 | | HDL≥1.15（mmol/L） | -0.39 | 1.56 | 0.68(0.37,1.25) | 0.2119 | | LDL＞2.16（mmol/L） | -0.36 | 1.56 | 0.70(0.40,1.23) | 0.2117 | | K＜3.5（mmol/L） | 0.81 | 7.55 | 2.25(1.26,4.00) | 0.0060 | | AFP＞7（ng/mL） | 0.53 | 2.90 | 1.70(0.92,3.12) | 0.0884 | | **Treatment and symptoms after diagnosis of PVT** | | | | | | Hepatic encephalopathy | 0.92 | 7.67 | 2.51(1.31,4.80) | 0.0056 | | Endoscope ligation | -0.24 | 0.11 | 0.79(0.19,3.25) | 0.7399 | | Abdominal infection | 1.09 | 10.27 | 2.98(1.53,5.82) | 0.0014 | | Blood transfusion | 0.74 | 6.76 | 2.09(1.20,3.64) | 0.0093 | | Abdominal surgery | 0.51 | 2.95 | 1.67(0.93,2.98) | 0.0858 | | Thrombolytic therapy | 0.04 | 0.02 | 1.05(0.58,1.87) | 0.8822 | | **The drugs of thrombolytic therapy** | | | | | | no drugs | REF |  |  |  | | anticoagulants | -0.10 | 0.41 | 0.90(0.66,1.24) | 0.5213 | | antiplatelets | -0.76 | 10.52 | 0.47(0.29,0.74) | 0.0012 | | **Anticoagulant drugs**  low molecular weight heparin | REF |  |  |  | | warfarin | -1.64 | 2.40 | 0.19(0.02,1.55) | 0.1217 | | **Anticoagulant duration（month）** | | | | | | ≤1 | REF |  |  |  | | 1-3 | -0.27 | 0.44 | 0.76(0.34,1.70) | 0.5066 | | ≥3 | -0.73 | 1.02 | 0.48(0.12,1.99) | 0.3136 | |  |  |  |
| --- | --- | --- | --- | --- | --- | --- | --- | --- | --- | --- | --- | --- | --- | --- | --- | --- | --- | --- | --- | --- | --- | --- | --- | --- | --- | --- | --- | --- | --- | --- | --- | --- | --- | --- | --- | --- | --- | --- | --- | --- | --- | --- | --- | --- | --- | --- | --- | --- | --- | --- | --- | --- | --- | --- | --- | --- | --- | --- | --- | --- | --- | --- | --- | --- | --- | --- | --- | --- | --- | --- | --- | --- | --- | --- | --- | --- | --- | --- | --- | --- | --- | --- | --- | --- | --- | --- | --- | --- | --- | --- | --- | --- | --- | --- | --- | --- | --- | --- | --- | --- | --- | --- | --- | --- | --- | --- | --- | --- | --- | --- | --- | --- | --- | --- | --- | --- | --- | --- | --- | --- | --- | --- | --- | --- | --- | --- | --- | --- | --- | --- | --- | --- | --- | --- | --- | --- | --- | --- | --- | --- | --- | --- | --- | --- | --- | --- | --- | --- | --- | --- | --- | --- | --- | --- | --- | --- | --- | --- | --- | --- | --- | --- | --- | --- | --- | --- | --- | --- | --- | --- | --- | --- | --- | --- | --- | --- | --- | --- | --- | --- | --- | --- | --- | --- | --- | --- | --- | --- | --- | --- | --- | --- | --- | --- | --- | --- | --- | --- | --- | --- | --- | --- | --- | --- | --- | --- | --- | --- | --- | --- | --- | --- | --- | --- | --- | --- | --- | --- | --- | --- | --- | --- | --- | --- | --- | --- | --- | --- | --- | --- | --- | --- | --- | --- | --- | --- | --- | --- | --- | --- | --- | --- | --- | --- | --- | --- | --- | --- | --- | --- | --- | --- | --- | --- | --- | --- | --- | --- | --- | --- | --- | --- | --- | --- | --- | --- | --- | --- | --- | --- | --- | --- | --- | --- | --- | --- | --- | --- | --- | --- | --- | --- | --- | --- | --- | --- | --- | --- | --- | --- | --- | --- | --- | --- | --- | --- | --- | --- | --- | --- | --- | --- | --- | --- | --- | --- | --- | --- | --- | --- | --- | --- | --- | --- | --- | --- | --- | --- | --- | --- | --- | --- | --- | --- | --- | --- | --- | --- | --- | --- | --- | --- | --- | --- | --- | --- | --- | --- | --- | --- | --- | --- | --- | --- | --- | --- | --- | --- | --- | --- | --- | --- | --- | --- | --- | --- | --- | --- | --- | --- | --- | --- | --- | --- | --- | --- | --- | --- | --- | --- | --- | --- | --- | --- | --- | --- | --- | --- | --- | --- | --- | --- | --- | --- | --- | --- | --- | --- |

HR, hazard ratio; CI, confidence interval; BMI, body mass index; GIB, gastrointestinal bleeding; SMV, superior mesenteric vein; PT, prothrombin time; INR, international normalized ratio; WBC, White blood cell; RBC, red blood cell; HB, hemoglobin; HCT, hematocrit; PLT, platelet count; CRP, C-reactive protein; ALB, albumin; ALT, alanine aminotransferase; AST, aspartate aminotransferase; TBIL, total bilirubin; DBIL, direct bilirubin; GGT, gamma-glutamyltransferase; SCR, serum creatinine; BUN, blood urea nitrogen; TC, total [cholesterol](javascript:;); TG, triglyceride; HDL, high-density lipoprotein; LDL, low-density lipoprotein; K, [kalium](javascript:;); AFP, alpha-fetoprotein.
